# Correction to ‘STRIDE: accurately decomposing and integrating spatial transcriptomics using single-cell RNA sequencing’

**DOI:** 10.1093/nar/gkac254

**Published:** 2022-04-07

In the Implementation of STRIDE from the Materials and Methods section, errors were introduced in the equation of calculating the topic-by-cell-type distribution during the production of the article (1). We apologize for these errors and wish to correct the equation as shown below.

Correct

$$\begin{equation*}\;{T_{kn}} = \frac{{{Z_{km}}{A_{mn}}}}{{\mathop \sum \nolimits_{i = 1}^M {A_{in}}}}\;\end{equation*}$$

to

$$\begin{equation*}\;{T_{kn}} = \frac{{\mathop \sum \nolimits_{m = 1}^M {Z_{km}}{A_{mn}}}}{{\mathop \sum \nolimits_{m = 1}^M {A_{mn}}}}\;\end{equation*}$$

The published article has been updated. These corrections do not affect the results, discussion, and conclusions presented in the article.
